# Planktonic foraminifera eDNA signature deposited on the seafloor remains preserved after burial in marine sediments

**DOI:** 10.1038/s41598-020-77179-8

**Published:** 2020-11-23

**Authors:** Inès Barrenechea Angeles, Franck Lejzerowicz, Tristan Cordier, Janin Scheplitz, Michal Kucera, Daniel Ariztegui, Jan Pawlowski, Raphaël Morard

**Affiliations:** 1 Department of Genetics and Evolution, University of Geneva, Boulevard d’Yvoy 4, 1205 Geneva, Switzerland; 2 Department of Earth Sciences, University of Geneva, Rue des Maraîchers 13, 1205 Geneva, Switzerland; 3 Jacobs School of Engineering, University of California San Diego, La Jolla, USA; 4 MARUM-Center for Marine Environmental Sciences, University of Bremen, Leobener Strasse 8, 28359 Bremen, Germany; 5 Institute of Oceanology, Polish Academy of Sciences, 81-712 Sopot, Poland

**Keywords:** Ecology, Molecular ecology, Marine biology

## Abstract

Environmental DNA (eDNA) metabarcoding of marine sediments has revealed large amounts of sequences assigned to planktonic taxa. How this planktonic eDNA is delivered on the seafloor and preserved in the sediment is not well understood. We address these questions by comparing metabarcoding and microfossil foraminifera assemblages in sediment cores taken off Newfoundland across a strong ecological gradient. We detected planktonic foraminifera eDNA down to 30 cm and observed that the planktonic/benthic amplicon ratio changed with depth. The relative proportion of planktonic foraminiferal amplicons remained low from the surface down to 10 cm, likely due to the presence of DNA from living benthic foraminifera. Below 10 cm, the relative proportion of planktonic foraminifera amplicons rocketed, likely reflecting the higher proportion of planktonic eDNA in the DNA burial flux. In addition, the microfossil and metabarcoding assemblages showed a congruent pattern indicating that planktonic foraminifera eDNA is deposited without substantial lateral advection and preserves regional biogeographical patterns, indicating deposition by a similar mechanism as the foraminiferal shells. Our study shows that the planktonic eDNA preserved in marine sediments has the potential to record climatic and biotic changes in the pelagic community with the same spatial and temporal resolution as microfossils.

## Introduction

Analyses of ancient DNA preserved in various archives have transformed our understanding of the evolution of species and ecosystems. Whilst earlier studies have concentrated on DNA extracted from taxonomically constrained samples (such as bones or frozen tissue), advances in high-throughput sequencing and bioinformatics now allow the analysis of ancient DNA extracted from sedimentary archives^1^, so called sedaDNA. The accumulation and preservation of sedaDNA buried in land and lake sediments have been subject to active research and interpretation^2^. However, studying the deposition of DNA on the ocean floor and its preservation in marine sediments is more complex because the DNA has to travel through a water column for several kilometers^3^. Unlike in the terrestrial environment, with pervasive transport of subfossil biomass from land, the largest portion of the marine sedaDNA is derived from planktonic community, which is dominated by microbes and protists^4^. After the death of the surface plankton, its DNA is subject to a transport through the water column, during which much of the associated organic matter is known to be consumed and respired^5^. This transport could take between 3 to 12 days depending on the size and morphology of test^6^. However, it remains unclear how exactly the planktonic eDNA, defined as the total DNA present in the environment after^7^, survives this transport, whether the degradation or transport are associated with sorting or lateral advection, and finally, whether the eDNA arriving at the seafloor is preserved in marine sediments without further distortion of its composition.

Despite the long exposure to degradation under oxic conditions during transport in the water column, and substantially lower concentration of organic matter on the seafloor, there is evidence that planktonic eDNA is preserved in marine sediments and contains exploitable ecological signal^8^. Earlier studies have shown sedaDNA preservation in marine sediments deposited under anoxia with unusually high amounts of organic matter preserved^9^, but later investigations indicate that sedaDNA can also be extracted from normal marine sediments, dominated by clastic or biogenic mineral fractions^10–12^. In addition, the low temperature of deep-sea water (0–4 °C) ensures a good preservation of sedaDNA^7,8^. Using planktonic foraminifera as a “Rosetta Stone”, allowing benchmarking of sedaDNA signatures by co-occurring fossil tests of these organisms, Morard et al. (2017)^9^ showed that the fingerprint of plankton eDNA arriving on the seafloor preserves the ecological signature of these organisms at a large geographic scale. This indicates that planktonic community eDNA is deposited onto the seafloor below, together with aggregates, skeletons and other sinking planktonic material. If this is true, sedaDNA should be able to record signatures of surface ocean hydrography, affecting the composition of plankton communities, with the same spatial resolution as the skeletal remains of the plankton. In addition, if the plankton eDNA is arriving on the seafloor in association with aggregates or shells, it is possible that it withstands the transport through the water column by fixation onto mineral surfaces. The same mechanism has been proposed to explain the preservation of sedaDNA in sediments^10–12^, implying that the flux of planktonic eDNA encapsulated in calcite test arriving on the seafloor is conditioned for preservation upon burial.

Planktonic foraminifera sedaDNA is an ideal proxy both “horizontally” to assess the spatial resolution of reconstructing past surface ocean hydrographic features and “vertically”, to unambiguously track the burial of its signal throughout the sediment column. Indeed, the flux of planktonic foraminifera eDNA should be proportionate to the flux of dead foraminiferal shells sinking to the seafloor, allowing independent benchmarking of the eDNA signal. eDNA is powerful tool to study ecosystem because it does not require direct taxonomic knowledge thus allowing to gather information on every organism present in a sample, even at the cryptic level. However, assignment of the eDNA sequences to known organisms is done via comparison with reference sequences (or barcodes) made available in public repositories or curated databases^13^. The taxonomy of planktonic foraminifera is well understood^14^ and barcodes exist allowing almost complete mapping of eDNA amplicons on the taxonomy based on foraminiferal test morphology^15,16^. Importantly, the composition of planktonic foraminifera communities is closely linked to surface hydrography and this signal is preserved by fossil tests deposited on the seafloor^17,18^. Since foraminiferal eDNA accumulated in the ocean sediment can be recovered, it could be used to analyze changes in planktonic and benthic communities over time^19–22^.

**Figure 1:**
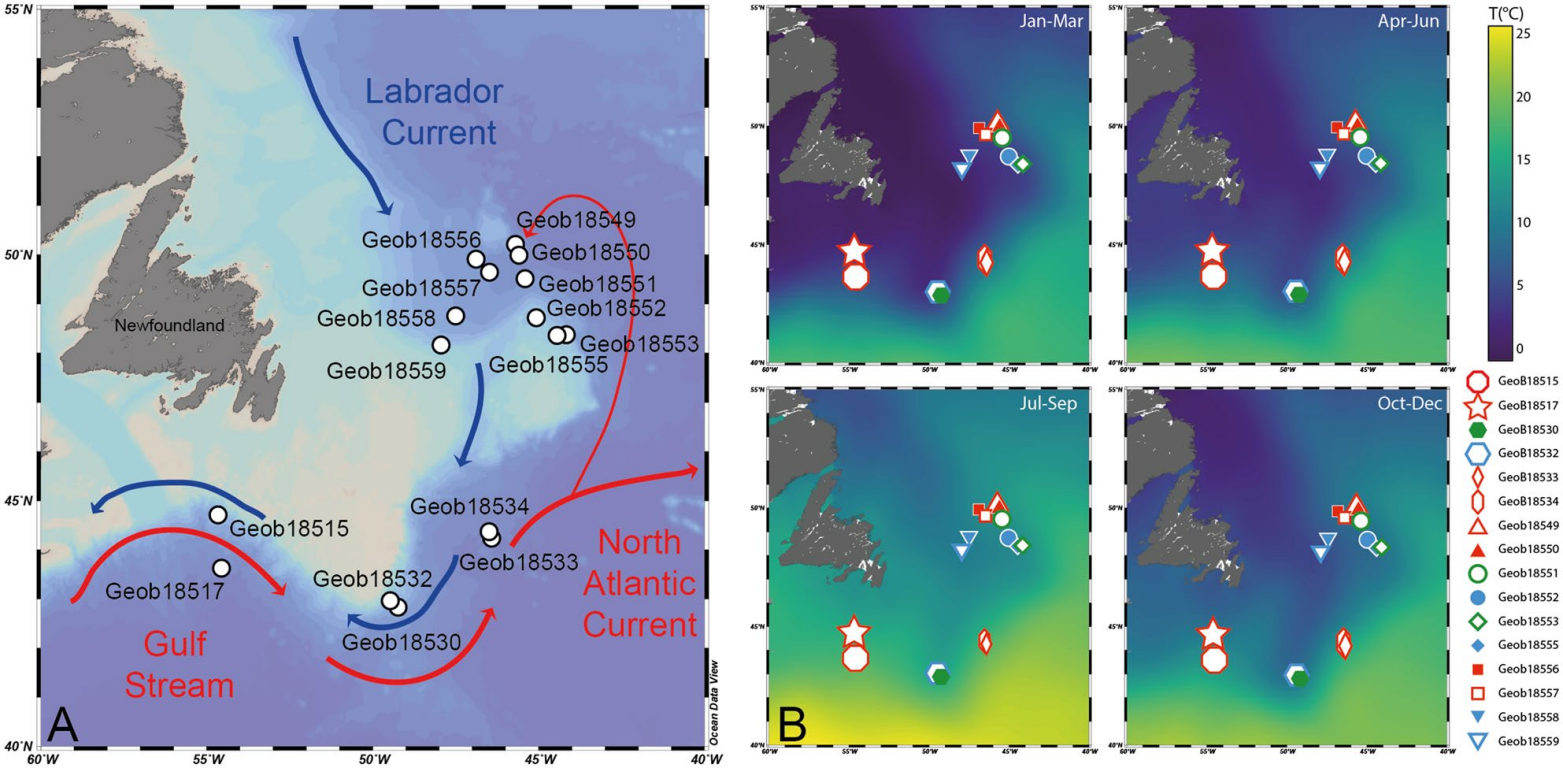
(**A**) Location of the analyzed cores. Red ascending arrows represent the Gulf Stream (GS) and North Atlantic Current (NAC), blue descending arrows the Labrador Current (LC). (**B**) Seasonal variability in sea-surface temperature observed in the sampling region. The data were extracted from the WOD13 database^57^. Open symbols represent locations where the cores were analyzed for micropaleontological and metabarcoding analysis, filled symbols indicate locations where only the core top was used for micropaleontological analysis. The maps were generated using Ocean Data View v.5.1.2^58^.

Here we take advantage of the planktonic foraminifera as a model system to investigate how planktonic foraminifera eDNA accumulates in sediments and to what extent the regional hydrographic features affecting the plankton are reflected in sedaDNA. We analyzed microfossil and molecular planktonic foraminifera assemblages in a series of short sediment cores, collected in the northwestern Atlantic, around the Grands Banks of Newfoundland, at the confluence of the cold Labrador and warm Gulf Stream Currents (Fig. 1). The southward Labrador Current (LC) exports cold and low salinity water from the Arctic Ocean in contrast to the northward Gulf Stream (GS) and North Atlantic Current (NAC) that bring warm and saline waters from the subtropical areas^23^, creating a steep and seasonally stable ecological gradient. The strength of the LC has influenced changes in climate during the Holocene by slowing down the Atlantic Meridional Overturning Circulation (AMOC)^24–26^ which exports warm water masses to the east Atlantic.

## Results

### Microfossil data

**Figure 2:**
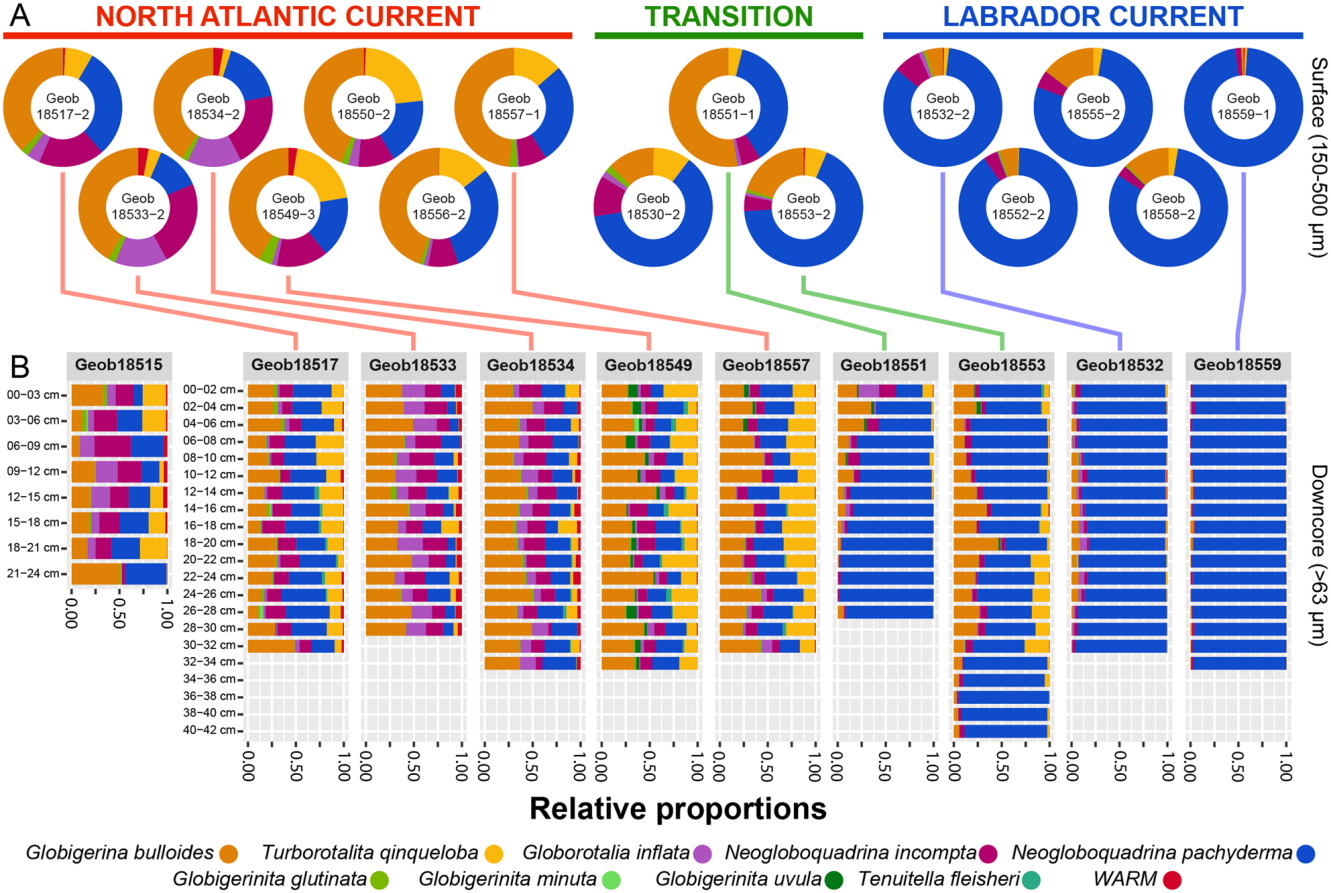
Census counts of planktonic foraminifera in the studied sediment cores (Fig. 1). (**A**) Counts performed in the surface sediments in the size fraction 150–500 µm at 15 locations. (**B**) Counts performed on the 10 cores selected for parallel census count and metabarcoding analysis. The cores are grouped according to the faunal signature of the regional hydrography preserved in the sediment. The graphs were generated with R^55^ using the ggplot2 package^59^.

Sediment samples were collected along the continental shelf off Newfoundland with multicorer (MUC) at 16 locations (Fig. 1) that allowed the recovery of short sedimentary cores of 24–42 cm (Fig. 2). The cores were sampled to carry out micropaleontological and metabarcoding analysis in parallel. We carried out first a census count of the size fraction 150–500 µm that is typical for micropaleontological analyses of 15 selected core-tops. Based on the obtained results, we carried out a census count of the samples of 10 cores representative of the compositional diversity at a size fraction in the fraction > 63 µm to capture compositional changes among small specimens and species. In all samples, 23 morphospecies of planktonic foraminifera could be identified, representing a mixture of polar, transitional and rare sub-tropical species. The five most common morphospecies were *Neogloboquadrina pachyderma* (polar), *Globigerina bulloides* (transitional), *Neogloboquadrina incompta* (transitional), *Turborotalita quinqueloba* (subpolar) and *Globorotalia inflata* (temperate) accounting for ~ 97% (83–100%) of the assemblages (Fig. 2). We also encountered the microperforate morphospecies *Globigerinita glutinata*, *Globigerinita uvula*, *Tenuitella fleisheri* and *Globigerinita minuta* that accounted for ~ 2% of the assemblages. Finally, we observed rare occurrences of morphospecies which are normally encountered in temperate to subtropical areas: *Globigerinoides ruber albus*, *Globorotalia hirsuta*, *Globorotalia menardii*, *Globigerina falconensis*, *Pulleniatina obliquiloculata*, *Trilobatus sacculifer*, *Globigerinoides conglobatus*, *Globigerinoides elongatus*, *Globigerinella calida*, *Globorotalia truncatulinoides*, *Neogloboquadrina dutertrei*, *Dentigloborotalia anfracta*, *Globigerinoides ruber ruber* and *Globigerinella siphonifera* that accounted for ~ 1% of the assemblages. These morphospecies were grouped into a category named “WARM” as they likely represent the advection of non-resident foraminifera by the Gulf Stream into the local community.

**Figure 3:**
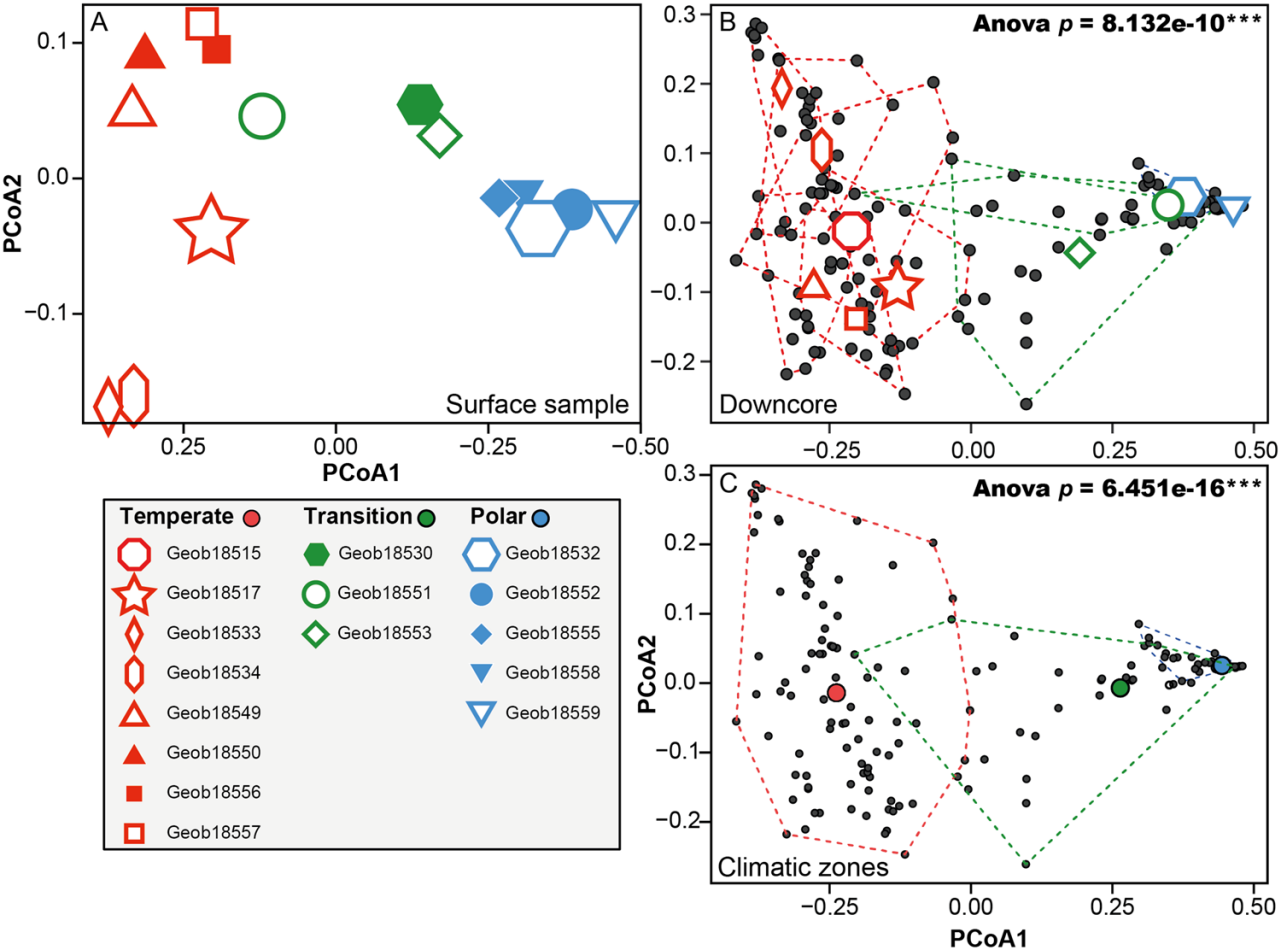
Principal coordinates analysis of the census counts of planktonic foraminifera carried out among (**A**) all surface samples, (**B**) all downcore samples with each site treated as a group and (**C**) all samples grouped by their hydrographic signature. The *p* value of the associated ANOVA is shown for (**B**) and (**C**) only because there is only a single surface sample per locality in (**A**). The graphs were generated with R^55^ using the ggplot2 package^59^.

Sedimentary assemblages record the westward retroflection of the NAC into the Labrador Sea that brings temperate and tropical species to the north of the Labrador Current (Fig. 1). Analysis of tests census counts in the surface samples of 15 sites (150–500 µm) that includes recent or subrecent specimens revealed that the planktonic foraminifera community at the sediment surface records the steep oceanographic gradient observed in the region (Figs. 2A, 3A). Indeed, we observed a strong polarization of the assemblages mostly driven by the relative proportion of *N. pachyderma,* a marker for cold temperatures, opposed to the transitional morphospecies *G. bulloides*, *T. quinqueloba, G. inflata, G. glutinata, N. incompta* and the WARM group. This compositional gradient largely follows the frontal zone between the NAC and the LC, including the counter-intuitive inversion of the temperature polarity in the Labrador Sea with samples collected to the South of Newfoundland having a “cold” signature (GeoB18530, GeoB18532) whilst samples collected Northeast off Newfoundland having a “warm” taxonomic composition (GeoB18549, GeoB18550, GeoB18551, GeoB18556, GeoB18557). This structure was largely conserved in the downcore analysis of the samples sieved at 63 µm (Figs. 2B, 3B,C). At sites GeoB18551 and GeoB18553 we observe higher compositional variability downcore (Fig. 3B), indicative of an episodically higher contribution of the “warm” species to these assemblages in the past, likely reflecting a shift of the front between the LSW and NAC influence in the past (Fig. 1). Surface and down-core sediments, and from both size fractions, could hence be classified into three zones reflecting (a) polar conditions dominated by *N. pachyderma*, (LC zone), (b) a mixed assemblage with higher proportion of the transitional species (transition zone) and (c) stronger advection of species of the WARM group due to higher influence of the NAC (NAC zone, Fig. 2A).

### Metabarcoding data

**Figure 4:**
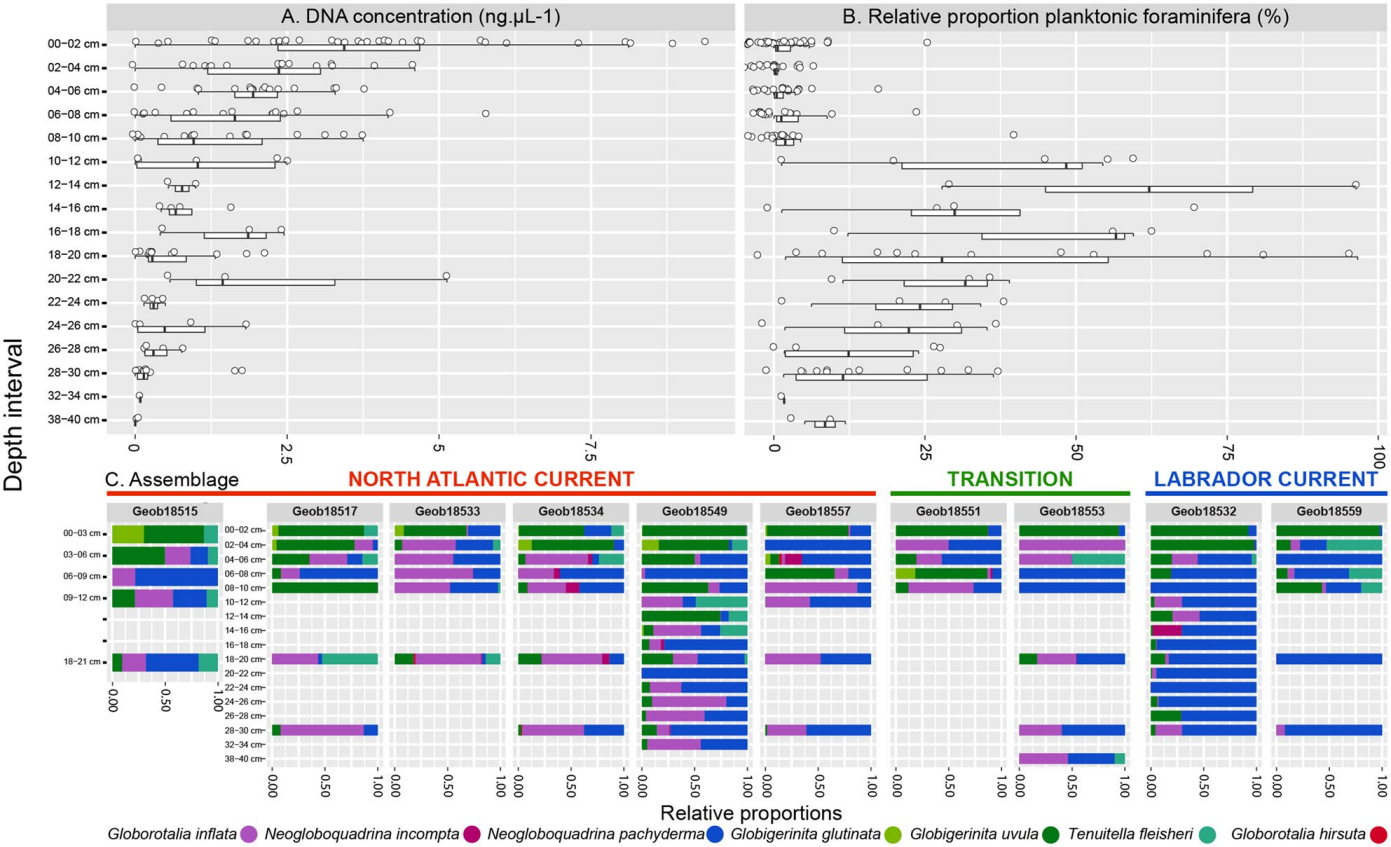
Results of metabarcoding analyses. (**A**) Box plot and jitter plot showing the DNA concentration in the sediment extract at all depths. (**B**) Proportions of planktonic and benthic foraminifera reads observed in the metabarcodes. (**C**) Relative proportions of reads assigned to individual species of planktonic foraminifera detected in the metabarcoding dataset. The graphs were generated with R^55^ using the ggplot2 package^59^.

We successfully amplified foraminifera metabarcodes from 167 samples. The total DNA extracted from sediment ranged from below the instrument detection limit to ~ 9 ng µl^−1^, and showed an overall decrease in concentration with depth (Fig. 4A). This decrease was mirrored by decreasing number of positive amplifications of foraminiferal DNA with increasing depth in the cores.

In total, 15,460,098 raw DNA amplicon reads were obtained from the 334 sequenced PCR products (2 replicates per sample) obtained from 167 samples. 8,306,918 reads were retained after quality filtering and clustered into 2467 OTUs. Of these, 1025 OTUs representing 7,908,878 reads occurred in more than three samples and were retained for subsequent analyses. From those, 10 OTUs accounting for 968,487 reads belonged to planktonic foraminifera and were assigned to the morphospecies *Neogloboquadrina pachyderma*, *Neogloboquadrina incompta*, *Globorotalia inflata*, *Globorotalia hirsuta*, *Globigerinita glutinata*, *Globigerinita uvula* and *Tenuitella fleisheri*. Three and two OTUs were attributed to the morphospecies G. uvula and *N. pachyderma* respectively as they were considered as intragenomic variants. No sequences of the two spinose planktonic foraminifera commonly encountered in the census counts *Globigerina bulloides* and *Turborotalita quinqueloba* were detected.

The ratio of planktonic to benthic foraminiferal reads changed with depth in the sediment. The relative proportion of planktonic reads was 3.5% on average in the top 10 cm of the cores (with rare outliers) but increased between 10 and 20 cm up to 96% of the total assemblages. Then, it dropped below 30% between 20 and 30 cm but remained higher than for the top 10 cm (Fig. 4B).

**Figure 5:**
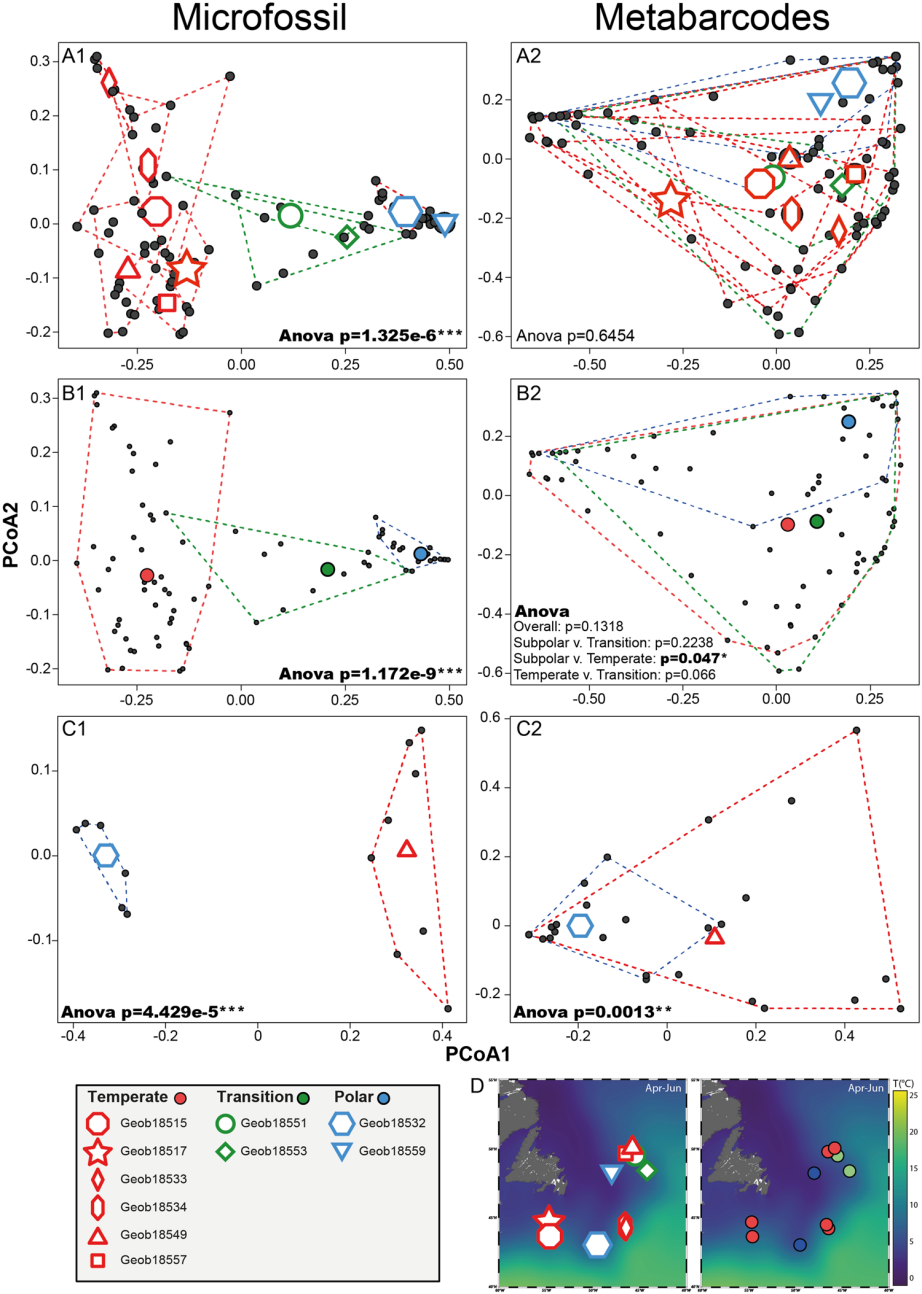
Comparison of principal coordinates analysis performed on the samples for which both morphological and metabarcoding analyses have been performed. The analyses have been performed with each site as a group (**A**), each hydrographic signature as a group (**B**) and only for the cores GeoB18532 and GeoB18549 (**C**) but without the layers between 0–4 cm that are dominated by *Globigerinita uvula*. The *p* value of the associated ANOVA is provided, and we provide each pairwise group for the (B2) analysis to show that the Polar vs Temperate is significant. Maps generated with Ocean Data View v.5.1.2^58^ showing the position of each core and the currents signature are provided (**D**). The graphs were generated with R^55^ using the ggplot2 package^59^.

In terms of the taxonomic composition, metabarcodes from the first four centimeters in all cores were dominated by reads assigned to the microperforate morphospecies *G. glutinata* and *G. uvula* (Fig. 4C). Further downcore, reads from these morphospecies became rare and the assemblage was dominated by *N. pachyderma* and *G. inflata.* Like in the micropaleontological samples, molecular assemblages of planktonic foraminifera in core GeoB18549 revealed substantial downcore variability, which was also present but less clearly developed in molecular assemblages in core GeoB18532 (Fig. 4C). Comparative analysis of microfossil and metabarcoding community showed an overall conservation of the same biogeographic patterns but with a larger degree of overlap in the metabarcoding data (Fig. 5). The significant difference observed between sites with microfossils (Fig. 5A1) is not conserved in molecular assemblages (Fig. 5A2). When comparing the composition of assemblages under the temperate (NAC), transition and polar (LC) currents, the significant difference is also not conserved overall among the metabarcodes, but the comparison of the sites under the temperate and polar regimes displayed a significant difference both in microfossil and metabarcoding data (Fig. 5B1,B2). To ensure that this observation was not only due to sampling size, we limited the comparison between the degree of the separation of the microfossils and metabarcoding datasets to the cores GeoB18532 (polar) and GeoB18549 (temperate) (Fig. 5C). The comparison of the distribution of the samples of the metabarcoding dataset returned a significant difference (p-value = 0.0013) despite the presence of an overlap in the metabarcode compositional data (Fig. 5C2), compared to the complete exclusion of the assemblages of the two cores observed in the microfossil assemblages (Fig. 5C1).

## Discussion

### Planktonic foraminifera eDNA preservation in the sediment

The observed decrease in total DNA concentration with sediment depth in all cores is consistent with a model of progressive degradation of sedaDNA over time (Fig. 4A). In this model, the higher concentrations at the surface would reflect the combination of the flux of eDNA from the plankton and DNA from living, sediment-dwelling organisms, including the benthic foraminifera, whose population density decreases with sediment depth. As a result, eDNA from living benthic foraminifera dominates the pool of foraminiferal sedaDNA in the top 10 cm of the sediment (Fig. 4). This 10 cm limit is entirely consistent with direct observations of depth range of benthic foraminifera in North Atlantic sediments identified as living by vital stains^27^, and by the depth of the bioturbated mixed zone in similar sediments^28^. The absence of living benthic foraminifera below 10 cm could also explain the remarkable sharp increase of the relative proportion of planktonic foraminifera sedaDNA immediately below this level. In the zone inhabited by living benthic foraminifera, eDNA from the living organisms is pristine and abundant, and thus more prone to be amplified and sequenced than the planktonic sedaDNA fraction^19^. Below the inhabited zone, all foraminiferal DNA is environmental and its amount reflects the flux of the involved populations. Since the flux of planktonic foraminifera is much higher than that of benthic foraminifera (as also seen in the concentration of their shells in the sediment with a ratio ~ 100:1), the composition of foraminiferal sedaDNA is skewed towards the plankton.

**Figure 6:**
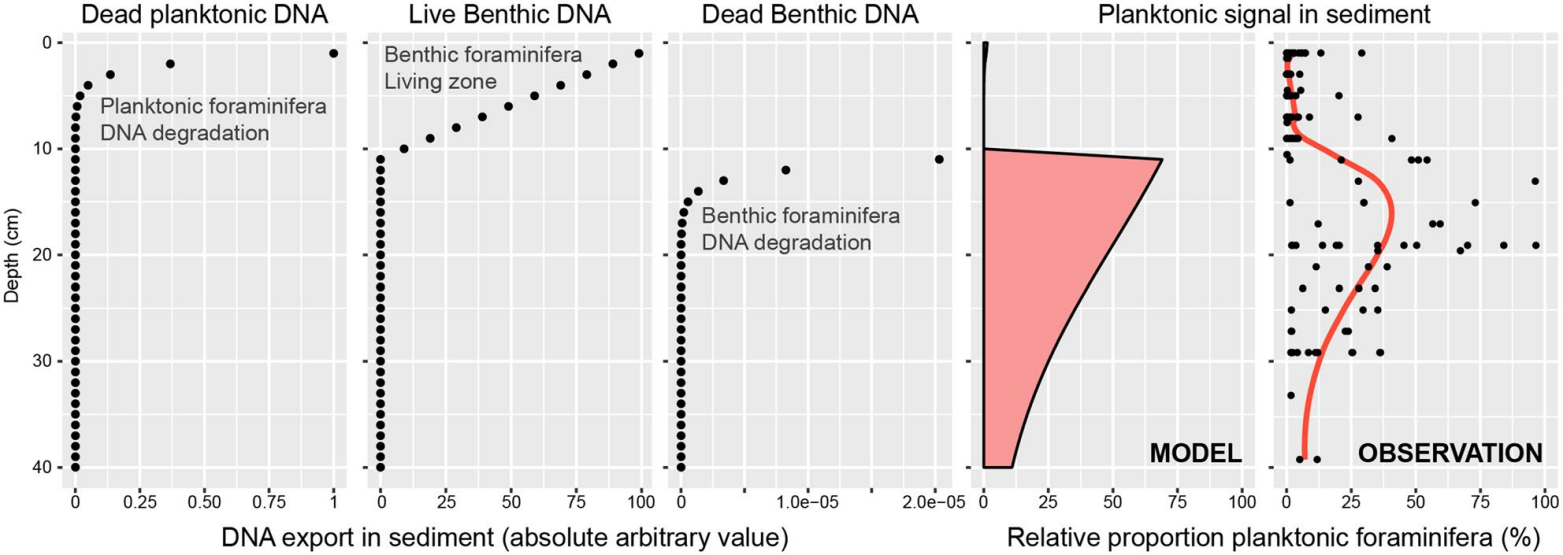
A conceptual model explaining the process of conservation of planktonic and benthic DNA with burial in the sediment. The flux of planktonic eDNA starts to degrade directly after the delivery onto the seafloor and is in competition with the DNA from living benthic foraminifera inhabiting the first 10 cm of the sediment. Below the living depth of benthic foraminifera, only their eDNA is preserved and therefore the relative abundance of planktonic foraminifera amplicons increases. The benthic DNA degrades at a lower rate than the planktonic DNA that leads to a decrease of relative abundance of planktonic DNA as observed in Fig. 2B. See “Discussion” for detailed explanation of the model. The graphs were generated with R^55^ using the ggplot2 package^59^.

To explain the observed pattern of relative abundance of planktonic and benthic reads, we considered that the benthic foraminifera community had an absolute abundance of 99 (arbitrary unit) at the surface and follows a linear decrease with depth to reach 1 at 10 cm which is the bottom of the inhabited zone in the sediment. Next to the living benthic foraminifera DNA, we considered a pool of planktonic eDNA exported from the surface that follows an exponential decay:

$$ N(t) = N_{0} e^{ - \lambda \tau } $$

where N_0_ is the initial abundance of planktonic foraminifera eDNA, λ is the decay constant and τ the mean lifetime, all set to 1. Below the inhabited zone (10 cm), we considered that there are no more living benthic foraminifera, and therefore its decay profile follows the same formula as for the planktonic foraminifera eDNA. However, we considered a lower decay constant for the benthic foraminifera DNA set to 0.9 because unlike the plankton, the decay profile of benthic DNA does not have to pass through the water column and is buried in the sediment directly with their cells. The resulting modeled relative proportions are shown on Fig. 6, indicating that the invoked process can at least theoretically reproduce the observed pattern.

In the deepest samples, the sedaDNA concentrations are low, in some samples even below the detection limit of the Qubit fluorometer used to quantify the DNA extract (Fig. 4A). However, the slope of both concentration and the relative abundance of benthic and planktonic eDNA appear to stabilize (Fig. 4B). The Holocene sedimentation rates on the continental slope off Newfoundland, including at some of the same locations where the studied cores were taken, vary between 15 and 25 cm/kyr^29^, indicating that the age of the oldest layers of the analyzed cores is likely > 2000 years. The consistent low concentration and consistent benthic/planktonic ratio in the oldest sedaDNA indicate that below the zone inhabited by the benthic foraminifera, there is no, or only limited, leakage of genomic DNA downcore, as shown already by previous studies^30,31^. Since we were able to recover a taxonomically consistent foraminiferal eDNA signature even in the samples with the lowest overall DNA concentration, a substantial part of this DNA must be present in amplifiable strands of at least 100–200 nucleotide length (microbarcode and flanking conserved region length). These results agree with previous studies showing good preservation of foraminiferal eDNA in a variety of marine sediments^19–21^. The observation of good preservation of longer DNA fragments is also consistent with studies of Pleistocene (older than 12,000 years) sedaDNA in marine sediments, which were based on PCR and sequencing of long barcodes^7,32,33^.

### Microfossil vs metabarcoding record

The morphospecies composition and relative abundances recorded in the microfossils, both at the surface and downcore, reflect fauna typical for the region^18^, mirroring in its composition remarkably well the steep hydrographic gradient along the continental margin off Newfoundland (Fig. 2). The metabarcoding community shows a similar geographical structure (Figs. 4, 5) and contains only taxa that were identified among the microfossils. This indicates that the sedaDNA both at the surface and downcore does not appear contaminated by long-range lateral transport and that no contamination likely occurred during laboratory handling of the samples. However, composition of the metabarcoding dataset differs from the census count dataset in two key aspects.

First, we observe systematic differences in the presence/abundance of some taxa that could be attributed to PCR-induced biases. The most striking is the absence in metabarcoding community of the spinose Globigerinidae^9,34^, such as *G. bulloides* and *T. quinqueloba* that are common among the microfossils in the studied material. This is due to a 1–3 nucleotide difference in the forward primer region between the spinose clade and other foraminifera*.* As a result, the recovered metabarcoding community contained no amplicons that could be assigned to spinose morphospecies. Considering the rapid evolution of the SSU rDNA gene in planktonic foraminifera^35^, it is difficult to design universal planktonic foraminifera primers for the hypervariable region 37F, which we deemed was the ideal target for studying ancient DNA because of its short length. Other foraminiferal metabarcoding studies also showed a primer bias, but were able to target all planktonic morphospecies^36,37^ using a longer 18S fragment, that we judged likely more difficult to amplify in sedaDNA samples.

Another PCR-related bias concerns the difference in species abundances between metabarcoding and microfossil data. This type of biases is due to either preferential PCR amplification or differences in gene copy number among species^38,39^. In our study this is best seen in the disproportionally high abundance of amplicons from the large *G. inflata* (Fig. 4), where we speculate that the species may have more gene copies per cell, since nothing indicates that the species should be preferentially amplified. Other than PCR biases or differential gene copy number, we see no evidence for systematic bias in sedaDNA preservation due to different sizes, shapes or thicknesses of the shells with which the DNA is likely associated. The most abundant amplicons were derived from OTUs assigned to the thick-walled medium-sized *N. pachyderma*, but many of the samples were dominated by amplicons from OTUs representing the thin-walled and tiny *G. uvula* and *T. fleisheri* in samples from the top 4 cm of the sediment (Fig. 4). We rule out primer and other sequence-related biases, that would result in a systematic preferential enrichment of this particular taxon at all depths.

It is also unlikely that this morphospecies, which has a broad ecological and geographic distribution^16^, was not recorded consistently in microfossils samples. The species is small, but its shells have been recovered at all depths, albeit at smaller quantities. Therefore, we retain the possibility that the high abundance of this species in surface metabarcoding datasets reflects a genuine signal. Indeed, it has been shown that the ongoing climate warming induces large latitudinal shifts in planktonic foraminifera communities globally^40^, and higher abundance of *G*. *uvula* in recent plankton samples from the North Atlantic has been noted by two independent studies^41,42^. We speculate that a potential recent increase of *G. uvula* in the studied region due to ongoing global change could in theory indeed be first observed in the eDNA on the sea floor. This is because the eDNA delivered during the last few years is the “freshest” and should amplify preferentially over older eDNA at the sediment surface. The most recent aDNA is likely completely mixed on short time scales over a few cm, explaining the abundance of *G. uvula* reads down to about 4 cm. Given the sedimentation rates reported in the region^29^, the top 4 cm of the studied sediments should represent the average deposition over ~ 200 years. This layer would be still numerically completely dominated by shells deposited before the recent plankton shift, but the aDNA in this layer would be biased towards the most recent signal. In this scenario, sedaDNA would act as a more sensitive recorder of recent changes in plankton flux than the fossil assemblage.

### Spatio-temporal patterns in community composition

Besides the two key differences described above and ascribed to PCR biases and bias due to amplification of the signal of a recent community shift, the composition of the metabarcoding community shows similar patterns of spatial and temporal variability as recorded by the fossil assemblage. Due to the lack of amplicons from spinose morphospecies, the difference between “warm” assemblages influenced by North Atlantic Current and “cold” assemblages deposited underneath the Labrador Currents are expressed more strongly in microfossil than metabarcoding data, but the spatial ordination of the sediment samples (surface and downcore) is similar (Fig. 5). The ANOVA performed on the microfossil assemblages returned highly significant values. The same analysis performed on the metabarcoding assemblages showed a larger degree of overlap between sites, but the pairwise ANOVA comparing metabarcoding and microfossil composition was still significant, showing essentially the same spatial ordination of the communities, irrespective of whether represented by microfossil counts or metabarcodes.

Importantly, these spatial patterns clearly remain preserved during burial. This is shown by the analyses of microfossil and metabarcode compositions in downcore samples from cores GeoB18532 and GeoB18549, representing deposition under colder and warmer conditions (Figs. 2, 4). Samples from both cores are clearly separated with respect to their microfossil assemblages and this separation remains preserved in the metabarcoding data, despite the lack of spinose amplicons (Fig. 4). Moreover, the downcore records also show similarities in the amount of compositional variance within each core between the microfossil and metabarcoding data. The microfossil community in the “cold” core GeoB18532 is strongly dominated by *N. pachyderma* (Fig. 2) and the metabarcoding data in this core is also dominated by this morphospecies (Fig. 4).

We selected the cores Geob18532 (polar) and GeoB18549 (temperate) for metabarcoding analysis on their entire length specifically because the microfossil assemblage polarity is opposite to the overall latitudinal gradient, with warmer fauna found to the North of Newfoundland, reflecting the NAC retroflection (Figs. 1, 2). The fact that the microfossil and metabarcoding communities both preserved the inverted regional features implies that both signals are congruent. Whereas foraminifera shells, made of calcite, sink to the seafloor within days, the DNA could, in theory, reside among the dissolved organic pool and its deposition could be delayed, allowing long-range mixing and advection. The fact that we observe highly congruent patterns, retrievable despite various sources of bias identified in the metabarcoding community, implies that the sedaDNA likely represents molecules delivered and preserved in association with the flux of planktonic foraminifera tests. Thus, sedaDNA preserved in marine sediments deposited under normal, oxic conditions, has the potential to record climatic and biotic changes in the pelagic community above the site of deposition with the same spatial and temporal resolution as microfossils. This conclusion certainly applies to the planktonic foraminifera, but can be likely extended to other planktonic taxa producing skeletal remains as well as those whose remains are deposited in the form of aggregates.

## Conclusion

The pool of sedaDNA can be considered as a reliable source of information that not only contains genomic remains, but also records paleoceanographic changes. We show that sedaDNA retains the fine scale regional oceanographic features, and we speculate that sedaDNA could detect rapid changes such as the recent oceanographic warming not yet recorded in the morphological samples. The same approach that we used for foraminifera could be applied to any group of plankton, even those that do not leave fossil remains and without requiring taxonomic expertise, provided that reference databases are complete enough to assign sedaDNA metabarcodes to known taxa. However, the metabarcoding approach has major pitfalls, since the PCR step can bias the community composition, and as it is shown in our case, omit an entire clade that is abundant in the samples. The careful design of primers is thus a prerequisite and when possible a parallel validation with fossil content of the samples should be mandatory.

## Material and methods

### Sampling

The sediment samples were collected along the continental shelf off Newfoundland with multicorer (MUC) at 16 locations (Fig. 1) during the MSM39 cruise^43^. The MUC allows the recovery of undisturbed sediment surface and the underlying 3–5 dm of the sediment. At each station, one small (Ø 5.6 cm) core was available for eDNA and micropaleontological sampling. The core was first gently pushed through the liner until the surface layer was at the level of the liner. The overlying water was removed using a clean syringe and the surface sediment was sampled with a sterile spatula and isolated into 1.5 mL tubes. Five replicates were taken for each MUC sampled. After the surface layer was sampled, the core was extruded in steps of 2–3 cm, the layer reaching above the liner was cut on the side and opened without touching the center. A sample from the pristine center of the slice was taken for DNA analyses with three to five replicates taken per layer using each time a different sterile spatula. The rest of the slice was used for micropaleontological analyses. The procedure was repeated until the bottom of the core was reached. The sediment samples for DNA analyses were frozen at − 80 °C after the end of the collection, which took less than half an hour per core. After each sampling the spatulas were rinsed clean, left for at least 30 min in a bath with diluted H_2_O_2_ and finally flamed using 100% ethanol before the next collection.

### Microfossil analysis

To assess the response of recent foraminifera community to the regional oceanographic settings, we selected 15 of the 16 locations for micropaleontological analyses of the core top samples. We subsampled the surface sediments and sieved them through 500, 150 and 63 µm size fractions. The fraction 150–500 µm was analyzed for census count of planktonic foraminifera of the surface samples. This size fraction was chosen to facilitate comparison with global datasets on planktonic foraminifera morphospecies abundances in seafloor sediments^18^. Based on the assemblage composition observed in the core tops (see “Results”), we selected ten cores representative of the assemblage variability across the ecological gradient to perform a comparison between morphological and molecular assemblages buried in the sediment. In the selected cores, we processed every available layer until the deepest sampled sediment (42 cm in core GeoB18553). For the 10 cores selected for comparison between microfossil and metabarcodes analyses, we counted foraminifera in the fraction > 63 µm to capture compositional changes among small specimens and small morphospecies, in order to provide data more comparable to the bulk metabarcoding datasets^16^.

The sediment samples were washed with freshwater and the residues were dried overnight at 40 °C. The dried samples were weighted and split using a microsplitter to obtain a representative aliquot containing 200–300 foraminifera for census counts. Planktonic foraminifera tests were identified and counted to morphospecies level following Schiebel and Hemleben (2017) and benthic foraminifera were counted as well. Metadata regarding the collection, wet and dry sediment weight processed, and census counts are reported in Table S1.

### eDNA extraction

We extracted the eDNA from all slices down to the 10 cm, from the 18–20 cm and 28–30 cm layers and in the longer core GeoB18553-3 also the layer 38–40 cm. In addition, in the cores GeoB18532-2 and GeoB18549-2, all available layers were processed, covering over 30 cm in both cores. We extracted between three to four replicates for the surface layers and between one and two replicates for the down core samples, resulting in a total of 167 sediment samples for metabarcoding analyses from 84 layers of the 10 cores. DNA extractions were carried out in batches of nine samples and one empty vial acting as a negative control in a clean dedicated sedaDNA room at the University of Geneva. Approximately 0.5 g of sediment of each subsample was extracted following the protocol of PowerSoil DNA Isolation Kit (MoBio) and the resulting DNA extracts were quantified with a fluorometer Qubit (Invitrogen) and are reported in Table S2. Gloves and disposable spatulas were changed and all surfaces were cleaned with bleach and RNase AWAY solutions between each sample and quantifications as well as other steps performed in high molecular load environments were done using single-use aliquots.

### PCR amplification and high-throughput sequencing

To enrich the foraminiferal signal in the DNA extractions, we carried out a PCR, making use of the relatively short and highly specific hypervariable region 37f (68–196 bp)^44^. The region was targeted using a combination of primers s14F1 (forward) and s15 (reverse), as previously described^45^. The primers were tagged with a unique combination of eight nucleotide identifiers attributed to each sample, allowing bioinformatic demultiplexing of the amplicons to their sample of origin. The tags’ combinations correspond to different nucleotides attached to the primers which allow a multiplex of samples and were designed according to a Latin square matrix^46^. To ensure a better PCR yield from the DNA extracts, we opted for the use of bovine serum albumin (BSA) (Thermo Scientific) and a polymerase from AmpliTaq Gold 360 Master Mix containing low-detection, heat-activated polymerase. The PCR mix contained: 15 µl of AmpliTaq Gold 360 Master Mix, 2 µl of BSA, 3 µl of combined primers at 0.2 M each, 10 ng of extracted DNA for samples having enough DNA, otherwise we added a maximum of extracted DNA as possible for those with low concentration, and H_2_O to complete 30 µl. To avoid contamination after preparing this mix, extracted DNA was added in a dedicated hood located in a separate room. For each PCR session 21 samples in duplicate together with 7 controls were processed, including 5 PCR blanks to ensure that both primers and PCR mix were clean and 2 extraction blanks to monitor DNA samples contamination in the extraction room. The PCR reaction was performed as follows: pre-denaturation at 94 °C during 1 min, then 60 cycles of denaturation at 94 °C for 30 s, annealing at 52 °C for 30 s and extension at 72 °C for 30 s, subsequently a final extension at 72 °C for 2 min. Aliquots of the PCR products were migrated on a 1.5% agarose gel for 20 min at 100 V, and quantified with Qubit fluorimeter (Invitrogen). The PCR products were pooled in equimolar mix with each duplicate located in a different pool to reach a total quantity of 100 ng of DNA. Each pool was purified using a High Pure PCR Cleanup Micro Kit (Roche) and quantified using the Qubit. One library was prepared for each pool following the instructions of the Illumina Truseq PCR-free Library Preparation kit. The resulting eight libraries were quantified by qPCR using KAPA Illumina Library Quantification and diluted to a final concentration of 4 nM. The diluted libraries were then pooled equimolar and sequenced on an Illumina Miseq system. The raw sequence data can be downloaded from the European Nucleotide Archive under BioProject PRJNA668798.

### Sequence data analysis

The raw sequences obtained from the libraries were processed as in^45^. The paired-end read pairs were quality-filtered by keeping only pairs having a mean quality score (phred score) above 30 and assembled with a minimum of 12 bases overlapping without mismatch. Those sequences were then demultiplexed based on the sequenced inline primer sequences, allowing a maximum of 2 mismatches to the reference tagged primer combinations. Chimeras were identified and removed with UCHIME 4.2^47^. The remaining sequences were further de-replicated to generate Individual Sequence Units (ISUs) as in^47^, each ISU was aligned using the Needleman–Wunsch (NW) algorithm against a multiple sequence alignment of foraminiferal species, and assigned to the consensus taxonomy of the sequences having the highest sequence identity level. ISUs without any alignment above 80% sequence identity with the reference database (see below) were left unassigned. To form OTUs, ISUs were pre-clustered based on their short 5′-end 37F hypervariable signatures (resolution described in Lecroq et al. 2010) and OTUs were delineated by average linkage clustering based on pairwise NW alignments distances and using thresholds defined for each pre-cluster based on the taxonomy of the ISUs to cluster.

In order to assign a taxonomy to OTUs, we assembled a custom reference multiple sequence alignment including planktonic and benthic taxa. We used the PFR^2^ v.1.0 database^15^ that includes planktonic foraminifera sequences only and added reference sequences of small planktonic foraminifera^37^ that were published after the release of PFR^2^. We merged the planktonic foraminifera reference sequences with those of benthic foraminifera species^48^ coming from NCBI GenBank. The taxonomy was structured into a 6-level hierarchical path that included the relevant level of the foraminifera taxonomy starting from the superorders^49^ until the genetic types for planktonic foraminifera^50,51^. The resulting alignment was trimmed to cover only the 37f region.

The OTUs were assigned using the assignment-table-vsearch module of the SLIM v0.4 web-application^52^ at 95% of similarity against the local reference multiple sequence alignment. Because of the high specificity of the selected region for foraminifera, all the recovered OTUs can be considered as derived from foraminifera^53^. OTUs that could not be assigned to the (almost) complete planktonic reference database were considered benthic. ISUs attributed to planktonic foraminifera by the assignment method were manually checked. Representative sequences of each planktonic foraminifera OTU were aligned with a selection of planktonic foraminifera reference sequences and only OTUs with clear similarity to the reference sequences were retained. The remaining ISUs were considered as benthic OTUs lacking close references in the multiple sequence alignment to be attributed with certainty. The result of the assignment of the ISUs are provided in Table S3.

The difference in number of reads recovered between libraries was normalized using the cumulative sum scaling method available on the metagenome-Seq Bioconductor package^54^ in R^55^. The cumulative sum scaling corrects the biases induced by differential sequencing depths and uses a zero-inflated Gaussian distribution mixture model that accounts for technical zero values resulting from under-sampling. In order to compare the community composition in both morphological and molecular data, we used the *betadisper* function in the *vegan* package^56^.

## Supplementary information

Supplementary Table S1.

Supplementary Table S2.

Supplementary Table S3.

## Data Availability

The raw sequence data can be downloaded from the European Nucleotide Archive under BioProject PRJNA668798 and the processed metabarcoding data and microplaeontological counts have been made available at PANGAEA under the accession 10.1594/PANGAEA.923417.
